# Workplace Violence Among Health Care Professionals in Public and Private Health Facilities in Bangladesh

**DOI:** 10.3389/ijph.2021.1604396

**Published:** 2021-12-31

**Authors:** Md. Shahjalal, Jeff Gow, Mohammad M. Alam, Tanvir Ahmed, Samar K. Chakma, Faroque M. Mohsin, Mohammad D. H. Hawlader, Rashidul A. Mahumud

**Affiliations:** ^1^ Department of Public Health, School of Health and Life Sciences, North South University, Dhaka, Bangladesh; ^2^ Research Rats, Dhaka, Bangladesh; ^3^ School of Business and Centre for Health Research, University of Southern Queensland, Toowoomba, QLD, Australia; ^4^ School of Accounting, Economics and Finance, College of Law and Management Studies, University of KwaZulu-Natal, Durban, South Africa; ^5^ Health, Nutrition and Population (HNP) Global Practice, The World Bank (Bangladesh), Dhaka, Bangladesh; ^6^ NHMRC Clinical Trials Centre, University of Sydney, Sydney, QLD, Australia; ^7^ Centre for Health Research, University of Southern Queensland, Queensland, QLD, Australia

**Keywords:** healthcare workers, prevention, physical violence, non-physical violence, workplace of violence

## Abstract

**Objectives:** The main objectives of this study were to examine the prevalence of workplace violence (WPV), its associated factors and explore the experiences of healthcare workers.

**Methods:** A hospital-based cross-sectional study design used a nationally representative sample of 1,081 healthcare workers covering eight administrative divisions of Bangladesh. Logistic regression analysis was employed to estimate the adjusted effect of independent factors on WPV among healthcare workers.

**Results:** Of the participants, 43% (468) experienced some form of WPV. Of those, 84% reported experiencing nonphysical violence, and 16% experienced physical violence in the past year. About 65% of victims claimed no action was taken to investigate the incident, and 44% reported no consequence for perpetrators. Four factors: being married (AOR = 1.63; CI: 1.12–2.39); public sector healthcare worker (AOR = 2.74; CI:1.99–3.76); working in an emergency department (AOR = 2.30; CI:1.03–5.12); and undertaking shift work (AOR = 1.52; CI: 1.10–2.11) were found to be significantly associated with WPV. One-third of the participants were worried about violence in their workplace.

**Conclusion:** WPV is highly prevalent among healthcare workers in Bangladesh. Formal guidelines for reporting and managing WPV are urgently needed at the individual, hospital, and national levels.

## Introduction

Workplace violence (WPV) encompasses both physical and nonphysical (verbal) violence. It is a growing public health concern among healthcare workers internationally, including in Bangladesh [1]. It is a key occupational hazard of healthcare workers. It is defined as any incident of a member of staff being abused, threatened, or assaulted on the grounds of their employment, including commuting to and from work, causing an implicit or explicit challenge to their safety, well-being, or health [2].

The World Health Organization (WHO) reported that violence in healthcare settings represents about a quarter of all violence in workplaces and that violence against healthcare workers is a global problem [3]. The WHO conducted a seven-country case study on WPV in the health sector and reported that more than 50% of responding workers experienced at least one incident of either physical or nonphysical violence in the preceding year: 37% in Portugal, 46% in Brazil, 54% in Thailand, 61% in South Africa, 67% in Australia, and 75% in Bulgaria [3]. A recent systematic review documented that the 12-months prevalence of any form of WPV was 61% globally among healthcare workers [4]. About 42% experienced nonphysical violence, for example, verbal abuse (57%), threats (32%), sexual advances (12%), while 24% reported physical violence. This review also indicated that 64% of Asian healthcare workers had experienced WPV: nonphysical 45% and physical 24% [4].

It is well known that violence occurs in all workplaces; however, healthcare workers are more prone to experience WPV than almost any other profession in both developed and developing countries [4, 5]. The prevalence of WPV among healthcare workers is high in Asian countries: 51% in Pakistan [6], 62% in China [7], and 63% in India [8]. These studies also estimated that verbal or nonphysical violence was more prevalent than physical violence among healthcare workers. In Bangladesh, violence against healthcare workers is not a new issue. An analysis of media reports suggests that 96% of reported violence cases were physical, 91% occurred in public health care settings, and 52% occurred in emergency departments [9]. Unfortunately, healthcare services in Bangladesh are experiencing severe shortages of skilled healthcare workers [10, 11].

Many studies have shown that nurses are more vulnerable than doctors and other healthcare workers [4, 6, 12] to WPV, while some studies found that doctors are most susceptible [8, 12]. Previous studies showed that a number of factors were associated with WPV in healthcare settings, including occupation, gender, age, marital status, healthcare level, healthcare sector, work schedule, and department [1, 2, 4–6, 8–10]. Most researchers reported that doctors [1, 4, 6], being male [4, 7], and younger age [2, 13] health workers have a higher risk of experiencing WPV. Additionally, healthcare workers who worked in the emergency department [1, 9, 10, 13], public hospital [1, 6, 9], tertiary healthcare facilities [1, 14], and shift work [4, 5, 13] were positively associated with WPV.

WPV has been associated with post-traumatic stress disorder symptoms such as being “super alert” and watchful, feeling chronic fatigue or being bothered by repeated memories of the incident, low performance, absenteeism and staff turnover, lower productivity, and motivation as well as professional dissatisfaction, leading to decreased quality of care for patients [3, 4]. WPV against healthcare workers has a devasting impact on victims’ psychological and social well-being [1, 4]. A significant portion of victims or those who witnessed WPV had some form of mental health consequence as a result. A recent study showed that around two-third of respondents had mental health problems after exposure to or witnessed violence [6]. After an episode of WPV, there are growing rates of missed workdays, burnout, job dissatisfaction, decreased productivity and ceasing employment [9, 13, 15]. In some cases of WPV, healthcare workers have protested to voice their opinion against offenders. This can lead to loss of workdays, reduced health services, and an increased burden on patients and the health care system [9, 13, 15].

Most reports on WPV and healthcare workers have appeared in the media through newspapers and electronic media in Bangladesh but without systematic research as to the actual prevalence and effects of WPV [9].

Therefore, this study sought to examine the prevalence of WPV, its associated factors and explore the experiences of healthcare workers. The study also aimed to examine preventive strategies and provide suggestions to policymakers.

## Methods

### Study Design and Participants

A hospital-based cross-sectional study of healthcare workers (doctors, nurses, others, e.g., midwives, laboratory technicians, administrative professionals) was conducted in primary, secondary, and tertiary level of healthcare settings, including public and private healthcare providers in eight administrative divisions of Bangladesh. The target population was healthcare workers who had patient contact. Personnel who met any of the following criteria were excluded: less than 1 year of work experience in the hospital, interns and trainees. Data were collected from November 2019 to March 2020.

### Survey Procedures

Sampling was stratified by healthcare facility type at the political administration level. Specifically, primary care facilities provide healthcare at the sub-district level (Upazilla health complex and below); secondary healthcare settings are usually located at the district level, while tertiary healthcare settings consist of medical colleges, specialist hospitals, and national hospitals, healthcare institutes, etc. Convenient sampling of primary, secondary and tertiary healthcare facilities across eight divisions in both public and private sectors was undertaken.

A structured questionnaire was used to collect socio-demographic information, healthcare settings, healthcare sectors, details of violence experienced in the past 12 months, problems and impacts encountered by the violent episode, and preventive measures. Healthcare workers on duty were invited to complete the questionnaire, and these included doctors, nurses, midwives, and laboratory technicians who had patient contact in their daily practices. Before conducting the survey, the study objectives, aims, methods, and benefits of this study were explained, and participants gave their written approval or not before proceeding.

The questionnaire was based on the WHO instrumental survey tool, previously used in Australia, South Africa, Portugal, Bulgaria, and Thailand [12]. In this study, that questionnaire was slightly adapted. The questionnaire comprised three sections [1]: Socio-demographic information and professional background [2]; Experience of WPV in the last 12 months, types and the number of instances, rehabilitation assistance to victims and their satisfaction with the assistance, reasons for underreporting or not reporting violence, and victim responses to and consequences for perpetrators [3]; Participant witnessing of violence in the previous year, anxiety and knowledge of WPV and preventive strategies. After voluntary consenting to participate, each respondent completed an anonymous questionnaire and returned it to the data collector. Respondents’ names and addresses were not required. Based on this procedure, 1,081 valid responses were collected (78.3% correct response rate).

### Operational Definition

Physical violence was defined as the application of force or action, including pinching, pushing, shoving, and spitting or kicking, with or without the use of weapons, as well as rape [3].

Non-physical violence was defined as verbal abuse, threats, bullying/mobbing, frightening action(s), and unwanted sexual advances [3].

### Statistical Analysis

We calculated descriptive statistics for demographic characteristics and frequency of exposure to WPV. Frequency distributions were calculated (in percentage points) for physical and nonphysical violence experienced by personnel in each professional category. In the analytical exploration, binary logistic regression analysis was used to determine which demographic and professional characteristics (including gender, marital status, age, experience, profession, department, shift work involved) associated experiencing physical or nonphysical violence. Based on only separated explanatory variables, an unadjusted regression analysis was performed. In the final model (adjusted), potential explanatory variables were only considered if any label of the covariate was statistically significant with a *p*-value at 5% or less in the unadjusted model. Statistical analyses were performed with SPSS v.25.

## Results

### Demographic and Professional Characteristics of the Respondents

A total of 1,081 healthcare workers participated in this study from all regions of Bangladesh (Table 1). The mean age of participants was 30.83 years (SD: 6.75; min:20, max:67). The majority of participants were doctors (64.0%), female (52.3%), <35 years of age (71.5%) and married (60.2%). Over half of the participants had experienced less than 6 years (61.4%), worked in tertiary healthcare settings (72.7%), for the public health sector (55.0%), and in fixed shift (51.8%). The spatial distribution of respondents was even across the nation.

**Table 1:** Participants’ demographic and professional characteristics, Bangladesh, 2020.

Participant characteristics	Number of participants (n)	Percentage of participants (%)
Profession		
Doctors	692	64.01
Nurses	285	26.36
Other healthcare workers	104	9.62
Gender		
Male	516	47.73
Female	565	52.27
Age		
<35 years	773	71.50
35–44 years	261	24.14
>44 years	47	4.34
Marital status		
Married	651	60.22
Unmarried	430	39.78
Level of healthcare setting		
Primary	168	15.54
Secondary	127	11.75
Tertiary	786	72.71
Type of healthcare sector		
Public	602	55.69
Private	479	44.31
Years of experience		
<6 years	664	61.42
6–10 years	211	19.51
11–15 years	121	11.20
16–20 years	44	4.07
>20 years	41	3.79
Working department		
General medicine	148	13.69
General surgery	149	13.78
Emergency	169	15.63
Intensive care	54	5.00
Pediatrics	88	8.14
Gynecology and obstetrics	95	8.79
Orthopedics	49	4.53
ENT (eye, nose and tongue)	30	2.78
Management	36	3.33
Other departments	263	24.33
Rotating shift work		
Yes	521	48.20
No	560	51.80
Workplace location		
Dhaka division	258	23.86
Chittagong division	144	13.32
Sylhet division	100	9.25
Khulna division	120	11.10
Rangpur division	137	12.67
Barisal division	98	9.06
Rajshahi division	123	11.37
Mymensingh division	101	9.34

**FIGURE 1:**
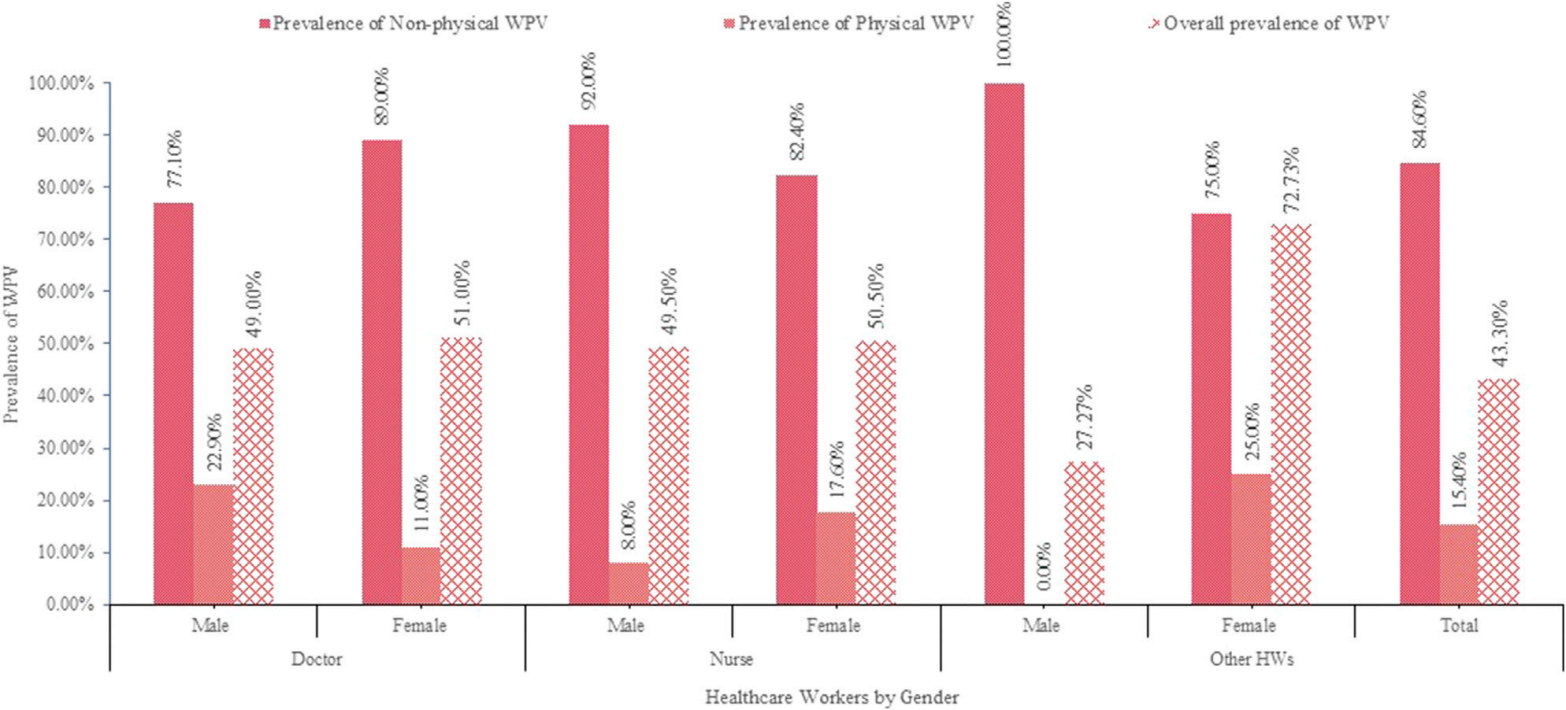
Prevalence of workplace violence (WPV) among healthcare workers, Bangladesh, 2020.

The prevalence of WPV varies by gender and profession (Figure 1). Overall, 468 (43.3%) incidents were reported, with 84.6% nonphysical and 15.4% physical. About 51.4% of doctors and 35.4% of nurses had exposure to some form of violence. Physicians were the most vulnerable to physical violence, while nurses were the most susceptible to nonphysical violence.

### Associations Between Violence Exposure and Respondents’ Characteristics

Table 2 shows the outcome of the multiple logistic regression model, which was used to assess the predictive factors for WPV. It was found to be significantly associated with profession, marital status, health care sector, specialized department, and shift work (*p* < 0.05).

**Table 2:** Multivariate adjusted and unadjusted odds for respondents’ exposure to violence, Bangladesh, 2020.

Participant characteristics	Prevalence of WPV	Unadjusted model		Adjusted model	
		OR	95% CI	AOR	95% CI
Profession					
Doctor	356 (51.4)	1.93	1.45–2.56	0.98	0.67–1.41
Nurse (=ref)	101 (35.4)	1.00	1.00	1.00	1.00
Other health workers	11 (10.6)	0.21	0.11–0.42	0.27	0.11–0.67
Gender					
Female	235 (42.6)	0.93	0.73–1.18	0.99	0.74–1.33
Male (=ref)	233 (44.0)	1.00	1.00	1.00	1.00
Age					
<35 years	274 (35.4)	0.34	0.18–0.62	0.94	0.16–5.39
35–44 years	165 (63.2)	1.07	0.56–2.02	1.33	0.26–6.84
>44 years (=ref)	29 (61.7)	1.00	1.00	1.00	1.00
Marital status					
Married	331 (50.8)	2.21	1.71–2.85	1.63	1.12–2.39
Unmarried (=ref)	137 (31.9)	1.00	1.00	1.00	1.00
Healthcare setting					
Primary	55 (32.7)	0.59	0.42–0.84	0.84	0.50–1.39
Secondary	59 (46.5)	1.06	0.73–1.54	0.96	0.60–1.53
Tertiary (=ref)	354 (45.0)	1.00	1.00	1.00	1.00
Healthcare sector					
Public	325 (54.0)	2.75	2.14–3.55	2.74	1.99–3.76
Private	143 (29.9)	1.00	1.00	1.00	1.00
Years of experience					
<6 years	205 (30.9)	0.26	0.13–0.49	0.50	0.08–3.23
6–10 years	124 (58.8)	0.82	0.41–1.64	0.84	0.14–5.15
11–15 years	86 (71.1)	1.42	0.67–2.99	1.06	0.17–6.50
16–20 years	27 (61.4)	0.91	0.38–2.20	0.62	0.12–3.08
>20 years (=ref)	26 (63.4)	1.00	1.00	1.00	1.00
Working department					
General medicine	60 (40.5)	0.61	0.29–1.26	0.64	0.29–1.41
General surgery	76 (51.0)	0.93	0.45–1.93	1.22	0.55–2.74
Emergency	123 (72.8)	2.39	1.14–4.99	2.30	1.03–5.12
Intensive care	26 (48.1)	0.83	0.35–1.93	0.85	0.33–2.15
Pediatrics	42 (47.7)	0.82	0.37–1.77	0.78	0.34–1.80
Gyne and obstetrics	37 (38.9)	0.57	0.26–1.23	0.56	0.24–1.30
Orthopedics	22 (44.9)	0.73	0.30–1.72	0.59	0.22–1.48
E.N.T. department	14 (46.7)	0.78	0.29–2.06	0.76	0.26–1.98
Other departments	49 (18.6)	0.20	0.09–0.42	0.37	0.17–0.79
Management (=ref)	19 (52.8)	1.00	1.00	1.00	1.00
Rotating shift work					
Yes	290 (55.7)	2.69	(2.10–3.45)	1.52	1.10–2.11
No (=ref)	178 (31.8)	1.00	1.00	1.00	1.00
Hosmer-Lesmeshow statistics (*p* value)				17.42 (*p* = 0.26)	

Compared to nurses, violence among doctors was higher (51.4%). The prevalence of WPV was significantly lower among other healthcare workers (AOR = 0.27; CI: 0.11–0.67). Participants who were married experienced a higher prevalence of WPV compared to those never married (AOR = 1.63; CI: 1.11–2.38). Participants from the public sector experienced 2.73 times higher WPV than their private-sector colleagues (AOR = 2.73; CI: 1.99–3.76).

Healthcare workers in emergency departments (AOR = 2.30; CI: 1.03–5.12) were more likely to have experienced WPV than those who worked in other departments. WPV was also higher among healthcare workers who were shift workers (AOR = 1.52; CI: 1.10–2.11).

### Perpetrators and Consequences

Regarding perpetrators, family members or relatives of the patients were the main perpetrators (73.1%), while 29.1% of cases involved the patient. Approximately 14.3% of victims reported being injured due to violence, while 22.4% had to leave work after being subjected to violence. Approximately 65% of victims claimed no action was taken to investigate the incident and 44.0% reported no consequences for the perpetrator(s) (Table 3).

**Table 3:** Distribution of study participants exposed to workplace violence, Bangladesh, 2020.

Variables	Number	Percentage
Perpetrators		
Patient	136	29.10
Relatives of the patient	342	73.10
Staff member	27	5.80
Supervisor or management	44	9.40
General Public	54	11.50
Injuries caused by violence		
Yes	67	14.30
No	401	85.70
After being a victim, take time off from work		
Yes	105	22.40
No	363	77.60
Action was taken to investigate the incident		
Yes	81	17.30
No	306	65.40
Don’t know	81	17.30
Consequences for the perpetrator		
None	206	44.00
Verbal warning	79	16.90
Care discontinued	16	3.40
Reported to police	16	3.40
Aggressor prosecuted	9	1.90
Do not know	142	30.30
Victims respond to the incident		
Yes	128	27.40
No	340	72.60
Incident was preventable		
Yes	282	60.30
No	186	39.70
Supports from employer		
Yes	94	20.10
No	374	79.90
Satisfaction with the way the situation was handled		
Yes	95	20.30
No	373	79.70
Reason for not reporting or talking about the incident		
It was not important	68	14.50
Felt ashamed	50	10.70
Felt guilty	22	4.70
Afraid of negative consequence	88	18.80
Fear of being fire from job	58	12.40
Did not know who to report	77	16.50
Others	105	22.40
Worried about violence in the current workplace		
Not worried at all	265	24.5
A little	300	27.8
Worried	244	22.6
Very worried	272	25.2
Procedures for the reporting of violence in the workplace		
Yes	422	39.0
No	265	24.5
Don’t know	394	36.4
Participated in any violence prevention training program		
Yes	251	23.2
No	830	76.8
Existing WPV prevention and control policies in the workplace		
Yes	118	10.9
No	382	35.3
Don’t know	581	53.7

### Reactions of Victims and Underreporting of WPV

Most of the victims (72.6%) stated that they did not react to the incident, while 60.3% believed it could have been prevented. Most victims (79.9%) claimed that their employer did nothing when the violence was reported to them, and 79.7% were not satisfied with the manner in which the situation was handled (Table 3).

WPV victims disclosed various types of reasons for not reporting incidents to the authorities. About 14.5% of victims thought it was unimportant, 16.5% said they did not know whom to report to, 18.8% were afraid of negative consequences, 10.7% felt ashamed, and 12.4% were afraid of being fired from their job. About 24.4% of participants were not worried about violence in their current workplace, while 27.8% were a little worried, 22.6% were worried, and 25.2% were very worried (Table 3).

Knowledge of preventive strategies and suggestions to prevent WPV About 24.5% of victims claimed no procedures for reporting the violent incidences, and 36.4% stated they had no idea about the process for reporting violence at their workplace. Participants were asked about any training or workshops on WPV they had undertaken, and 76.8% replied that they had not taken part in any training or workshop to deal with WPV. Over half of respondents (53.7%) stated no existing WPV prevention and control policies in their workplace (Table 3).

**FIGURE 2:**
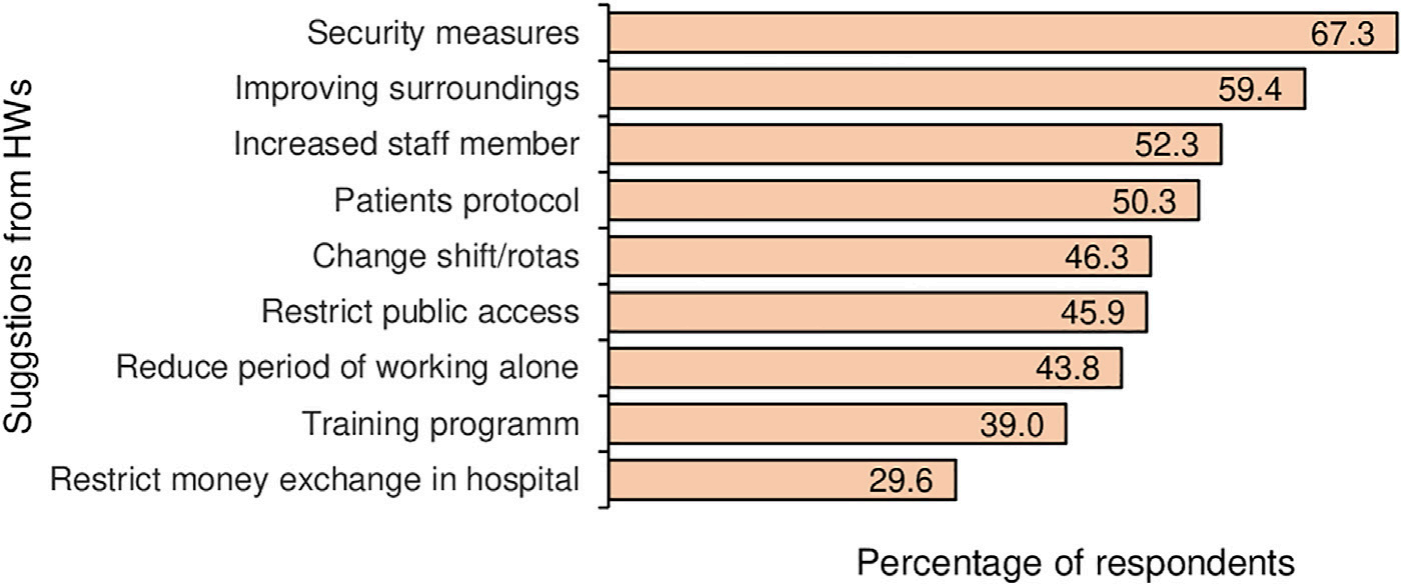
Suggestions from healthcare workers (HW) to prevent workplace violence (multiple responses), Bangladesh, 2020.

Participants were also asked about measures that could be taken to prevent violence. All participants could mark more than one choice. Measures that could be taken to avoid the violence include security measures 67.2% (727), improving surroundings 59.4% (642), increased staff members 52.2% (565), and patient protocol 50.3% (544). They also suggested that training programs, reduced time working alone, shift changes, restricting public access, and restricting money exchange in hospitals could also be effective (Figure 2).

## Discussion

This study investigated workplace violence experiences among healthcare workers (doctors, nurses, midwives, medical technologists, administrative staff) in Bangladesh and its characteristics. The analysis revealed the overall prevalence of WPV and differences in prevalence among various categories of healthcare workers. Moreover, the distribution of physical and nonphysical violence, associated factors, and potential preventive strategies was analyzed.

This study obtained a 43.3% prevalence of WPV among healthcare workers, of which 84.6% were non-physical, and the remaining 15.4% was physical. The prevalence is lower than a study conducted in China (49.2%) [17] but higher than in India (40.8%) [18]. A Turkish study of doctors identified a very high 78.1% prevalence of WPV in the emergency department [16]. The current study has also revealed that respondents from the emergency department (72.8%), followed by management (52.8%) and general surgery (51%), were more likely to have experienced violence than those who worked in other departments. There have been increasing workplace violence trends, as previously reported that in Bangladesh, 91% of violence took place in public healthcare settings [9]. Studies conducted worldwide identified quite diverse prevalence rates among the various types of health care professionals and at multiple levels (primary, secondary, tertiary) [14, 19, 20]. In this study, a higher prevalence of WPV among doctors compared to nurses was observed. However, WPV was very low (10.6%) among other healthcare workers. The prevalence was also relatively low in primary health care settings compared to secondary and tertiary levels.

Doctors in an emergency, psychiatrists, and those involved with primary care are at increased risk of violent acts from patients and families. Emergency departments, intensive care units, and post-surgical wards are the most common settings for violence [19, 20]. Working in an emergency department with a high patient admission rate increases the probability of experiencing WPV [16]. There were statistically significant differences in the experience of violence who worked at public and private hospitals. A study conducted in Palestine showed a tendency for those who worked in public hospitals to be more likely to experience violence [21]. Further, respondents who were shift workers were four times more likely to have experienced violence than those who did not work shifts [22].

There are often difficulties with relations between healthcare workers and their patients in Bangladesh, as multiple newspaper reports collated and published recently can attest [23]. Most perpetrators of violence were relatives of the patient. An Indian study also identified similar findings as perpetrators were visitors/relatives (48%), patients (38%), and co-workers (14%) [21].

Non-reporting of violence is a concerning issue, mainly due to the lack of policy or procedure and management support, having previous experience of no action taken, and fear of the consequences. The results identified not knowing whom to report to as the main reason behind non-reporting. The majority of the victims took no action to investigate the incidents, and 36.6% had no idea about the consequences for the perpetrator(s).

The study results confirm that among the suggested measures, security measures were the most commonly implemented. However, these measures were reactive rather than proactive and tackled a particular risk (physical violence) rather than WPV as a whole. In light of international and national experiences, it is only with comprehensive preventive measures and penalties that actions against WPV at hospitals can be practical.

As outlined in the international labor organization (ILO) convention (No. 190) and recommendation (No. 206), “Governments should adopt legislation requiring employers to secure adequate protection against workplace violence and harassment.” [24]. However, no national guidelines in Bangladesh ensure employers’ legal responsibility to provide a safe, decent and healthy working environment for employees, where protection of their legitimate rights is enforced. The interests of health workers and, in particular, their safety is not being prioritized by specific government legislation.

The outlined WPV preventive measures are widely accepted as comprehensive measures to address workplace the risk factors of WPV. Therefore, the following are suggestions for wide measures to combat WPV in hospitals in Bangladesh. *First*, create a positive culture to combat violence. To create a harmonious doctor-patient relationship, respect, tolerance, gender sensitivity, equality, collaboration, and care should be practiced, and no form of WPV should be tolerated. *Second*, organizational interventions should be conducted whereby hospitals need to invest in human resource development. Improving staff or patient rations to reduce staff shortages will minimize time pressure on health workers. Effective organization of workloads can reduce the number of consecutive night shifts and the long working hours experienced.

*Third*, implement interventions to optimize service delivery to reduce waiting times, design comfortable and convenient waiting areas, and design escape doors for high-risk departments (such as emergency) staff. It is crucial to limiting public access, including security checks on visitors, and provide safe areas for staff (such as changing rooms). *Fourth*, hospitals should be required to provide victims with medical treatment, psychological counseling, and financial compensation, while perpetrators should be punished under the law. *Fifth*, develop and introduce practical measures for prevention and control, such as measuring the prevalence of violence, the impact of violence, and undertaking risk assessments.

### Strengths and Limitations

This survey is the first comprehensive study of WPV against healthcare workers and revealed the WPV situation in healthcare settings in Bangladesh. In this study, relatively a large sample size was obtained, considering the total number of healthcare workers in Bangladesh. The study results can contribute to developing appropriate policy and strategies on WPV against healthcare workers and serve as the basis for future research in the country.

This study has several limitations. *First*, this study was completed in response to an open invitation; thus, it might have been completed mainly by healthcare workers who had been subjected to violence in the past and therefore are more sensitive to this issue. Data were collected retrospectively; these methods rely on the respondents’ ability to recall events in the past year, which could result in recall bias. *Second*, a convenience sampling design was used in this study, so the results cannot be generalized. *Finally*, the survey was not tested and validated before this study.

### Conclusion

This study has outlined the prevalence of WPV among healthcare workers in Bangladesh, and the results indicate that these healthcare workers are vulnerable to WPV. Several potential associated factors of WPV, such as profession, marital status, healthcare sector, specialized department, and shift work was observed. Some critical factors, such as the reluctant attitudes of employers and employees regarding WPV, which include underreporting by employees, lack of knowledge among healthcare workers, low job security, and inefficient action by authorities, are some of the primary reasons behind the burden of WPV. Considering our findings, this issue cannot be ignored, especially from the point of view of occupational health and safety. To reduce WPV and create safe working environments, we recommend developing preventive safety policies, procedures, and prevention training. Further research is needed to understand how to reduce WPV against healthcare workers.
